# Development of an Immune-Related Prognostic Index Associated With Glioblastoma

**DOI:** 10.3389/fneur.2021.610797

**Published:** 2021-05-19

**Authors:** Zhengye Jiang, Yanxi Shi, Wenpeng Zhao, Yaya Zhang, Yuanyuan Xie, Bingchang Zhang, Guowei Tan, Zhanxiang Wang

**Affiliations:** ^1^Department of Neurosurgery, Xiamen Key Laboratory of Brain Center, The First Affiliated Hospital of Xiamen University, Xiamen, China; ^2^School of Medicine, Institute of Neurosurgery, Xiamen University, Xiamen, China; ^3^Department of Cardiology, Jiaxing Second Hospital, Jiaxing, China

**Keywords:** TCGA, glioblastoma, tumor microenvironment, immune scores, overall survival, CGGA, drugs

## Abstract

**Background:** Although the tumor microenvironment (TME) is known to influence the prognosis of glioblastoma (GBM), the underlying mechanisms are not clear. This study aims to identify hub genes in the TME that affect the prognosis of GBM.

**Methods:** The transcriptome profiles of the central nervous systems of GBM patients were downloaded from The Cancer Genome Atlas (TCGA). The ESTIMATE scoring algorithm was used to calculate immune and stromal scores. The application of these scores in histology classification was tested. Univariate Cox regression analysis was conducted to identify genes with prognostic value. Subsequently, functional enrichment analysis and protein–protein interaction (PPI) network analysis were performed to reveal the pathways and biological functions associated with the genes. Next, these prognosis genes were validated in an independent GBM cohort from the Chinese Glioma Genome Atlas (CGGA). Finally, the efficacy of current antitumor drugs targeting these genes against glioma was evaluated.

**Results:** Gene expression profiles and clinical data of 309 GBM samples were obtained from TCGA database. Higher immune and stromal scores were found to be significantly correlated with tissue type and poor overall survival (OS) (*p* = 0.15 and 0.77, respectively). Functional enrichment analysis identified 860 upregulated and 162 downregulated cross genes, which were mainly linked to immune response, inflammatory response, cell membrane, and receptor activity. Survival analysis identified 228 differentially expressed genes associated with the prognosis of GBM (*p* ≤ 0.05). A total of 48 hub genes were identified by the Cytoscape tool, and pathway enrichment analysis of the genes was performed using Database for Annotation, Visualization and Integrated Discovery (DAVID). The 228 genes were validated in an independent GBM cohort from the CGGA. In total, 10 genes were found to be significantly associated with prognosis of GBM. Finally, 14 antitumor drugs were identified by drug–gene interaction analysis.

**Conclusions:** Here, 10 TME-related genes and 14 corresponding antitumor agents were found to be associated with the prognosis and OS of GBM.

## Introduction

Glioblastoma (GBM) is a highly aggressive brain tumor with poor prognosis (1). The 1- and 5-year survival rates of GBM are estimated to be 35.7 and 4.7%, respectively, while its average overall survival (OS) time is 12–18 months (2). The Cancer Genome Atlas (TCGA) is a valuable resource used for the classification and discovery of large cancer gene expression datasets (3, 4). GBM is classified into four subtypes: proneural, neural, classic, and mesenchymal (5). In 2016, the WHO updated the classification of GBM into three subtypes based on molecular and histological features: (1) isocitrate dehydrogenase (IDH) wild type, (2) IDH mutant, and (3) not otherwise specified (NOS) (unspecified) (6). Several genes and transcription factors have been shown to play a critical role in the occurrence, development, and evolution of GBM (7, 8). Other scholars have demonstrated that the tumor microenvironment (TME) strongly influences gene expression in tumor tissues, thus having a notable impact on the clinical outcomes of cancers (9–14). The TME refers to the environment around a tumor and is mainly composed of immune cells, endothelial cells, mesenchymal cells, inflammatory mediators, and stromal cells (15, 16). In tumors, immune and stromal cells are the two main non-tumor constituents with diagnostic and prognostic potential.

Various tools have been developed to analyze the molecular composition of the TME in TCGA gene expression datasets (14, 17). For example, the estimation of stromal and immune cells in malignant tumor tissues using expression data (ESTIMATE) scoring algorithm estimates the number of stroma and immune cells in tumors (14). This algorithm predicts non-tumor cell infiltration by analyzing the expression patterns of particular genes in immune and stromal cells and also calculates their scores. ESTIMATE has been applied to study various cancers, including breast (18), prostate (19), and colon cancer (20). However, the significance of immune and stromal scores in GBM has not been established.

Currently, GBM is difficult to treat because of several challenges, such as the blood–brain barrier, tumor heterogeneity, drug efflux pumps, and glioma stem cells (21, 22). Therefore, it is important to find new druggable targets for the treatment of GBM.

Herein, we calculated the immune and stromal scores of GBM cohorts downloaded from TCGA database using the ESTIMATE algorithm (14). An array of TME-associated genes that predict poor outcomes in GBM patients were found. Finally, the Chinese Glioma Genome Atlas (CGGA) GBM cohort was used to validate the prognostic performance of the genes. Drugs targeting the genes were identified in the Dgidb database.

## Materials and Methods

### Database

The transcriptome profiles of GBM patients were obtained from TCGA database (https://tcga-data.nci.nih.gov/tcGA/). The gene expression data of GBM were determined using Agilent G4502A072 (Sep 08, 2017). Clinical data, including gender, tissue type, prognosis, and survival rates, were retrieved from TCGA database. Immune and stromal scores were calculated using the ESTIMATE algorithm (14). The gene expression profiles of GBM patients were downloaded from the CGGA database (http://www.cggA.org.cn/). The RNA sequencing of samples from diffuse gliomas was performed using the Agilent Whole Human Genome array. The survival and prognosis data were downloaded from the CGGA database.

### Identification of Differentially Expressed Genes

The datasets were analyzed using the LIMMA package (23). Fold change ≥1.5 and adjusted *p* ≤ 0.05 were set as the cutoff thresholds for selection of differentially expressed genes (DEGs).

### Construction of a Protein–Protein Interaction Network and Module Analysis

Protein–protein interaction (PPI) networks were constructed using STRING v11.0. The full gene list was uploaded to the database. Disconnected nodes were excluded from the resulting network (24). The PPI network was visualized using Cytoscape (version 2.8.1) (25).

### Overall Survival Analysis

The association of the hub genes with the OS rate was determined using survival data of GBM patients obtained from TCGA-BRCA database.

### Gene Ontology and Kyoto Encyclopedia of Genes and Genomes Pathway Analyses

Gene ontology (GO) is a tool used to predict the biological processes (BPs) of gene. The GO terms analysis included BPs, molecular function (MF), and cellular composition (CC) (26, 27). Kyoto Encyclopedia of Genes and Genomes (KEGG) is an open-access resource for analyzing the biological pathways of genes. These tools were used to determine functions and pathways associated with the prognostic value genes (28). *p* ≤ 0.05 was considered statistically significant.

### Drug–Gene Cross-Talk and Functional Analysis of Potential Genes

The Drug Gene Interaction Database (DGIdb) was used to determine potentially druggable targets for the mutated and altered genes (29).

### Statistical Analysis

All statistical analyses were performed using R/BioConductor (version 3.6.3) with two-tailed *p*-values. For Cox regression analyses, survival data that show the state of survival and the time of follow-up were considered the dependent variable, whereas continuously expressed values of the 10 RNAs were considered as the independent variables. Kaplan–Meier (KM) plots and receiver operating characteristic (ROC) curves for the prognosis model were constructed using R (survival, survminer, and ggplot2 packages). The Cox proportional hazards regression model was applied to determine OS, and log-rank test was used to compare differences in OS. *p* < 0.05 was considered statistically significant.

## Results

### Immune and Stromal Scores Are Associated With Glioblastoma Subtypes and Survival Outcome

**Figure 1:**
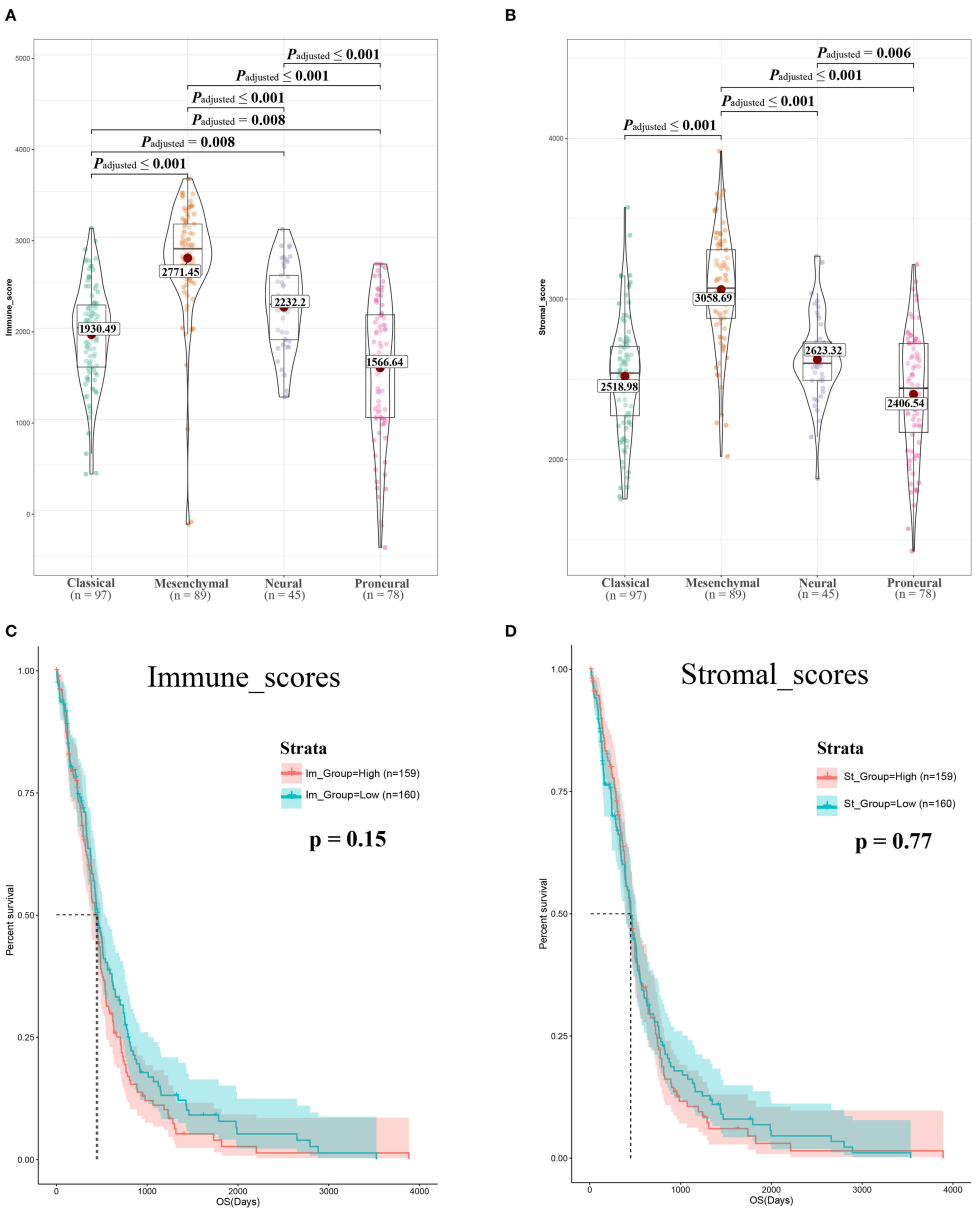
Immune scores and stromal scores are associated with glioblastoma (GBM) subtypes and their overall survival. **(A)** Distribution of immune scores of GBM subtypes. Violin plot shows that there is significant association between GBM subtypes and the level of immune scores (*n* = 309, *p* < 0.001). **(B)** Distribution of stromal scores of GBM subtypes. Violin plot shows that there is significant association between GBM subtypes and the level of stromal scores (*n* = 309, *p* < 0.001). **(C)** GBM cases were divided into two groups based on their immune scores: as shown in the Kaplan–Meier survival curve, median survival of the low-score group is longer than high-score group; it is not statistically different as indicated by the log rank test; *p*-value is 0.15. **(D)** GBM cases were divided into two groups based on their stromal scores: the median survival of the low-score group is longer than the high-score group; similarly, it is not statistically different as indicated by the log rank test *p* = 0.77.

Gene expression profiles of 309 GBM cases initially diagnosed between 1989 and 2010, and their associated clinical data, were downloaded from TCGA dataset. Of the 309 GBM cases, 122 (39.5%) were female and 187 (60.5%) were male. Using the ESTIMATE algorithm, we calculated the stromal scores, which ranged from 1,430.1 to 3,920.55, and immune scores, which ranged from −405.52 to 3,640.43 (Figures 1A,B). The mesenchymal cell subtype had the highest immune and stromal scores, indicating that immune and stromal scores can be used for subtype classification.

To determine the correlation between OS and the two scores, 309 GBM cases were divided into high- and low-score groups based on the immune and stromal scores. KM analysis revealed that patients with high immune scores had lower OS than those with low immune scores (442 vs. 394 days, log-rank test *p* = 0.15, Figure 1C). Notably, patients with high interstitial scores had shorter median OS (442 vs. 422 days, log-rank test *p* = 0.77, Figure 1D) than patients with low interstitial scores, although the difference was not significant.

### Association of Gene Expression Profiles With Immune Scores and Stromal Scores in Glioblastoma

**Figure 2:**
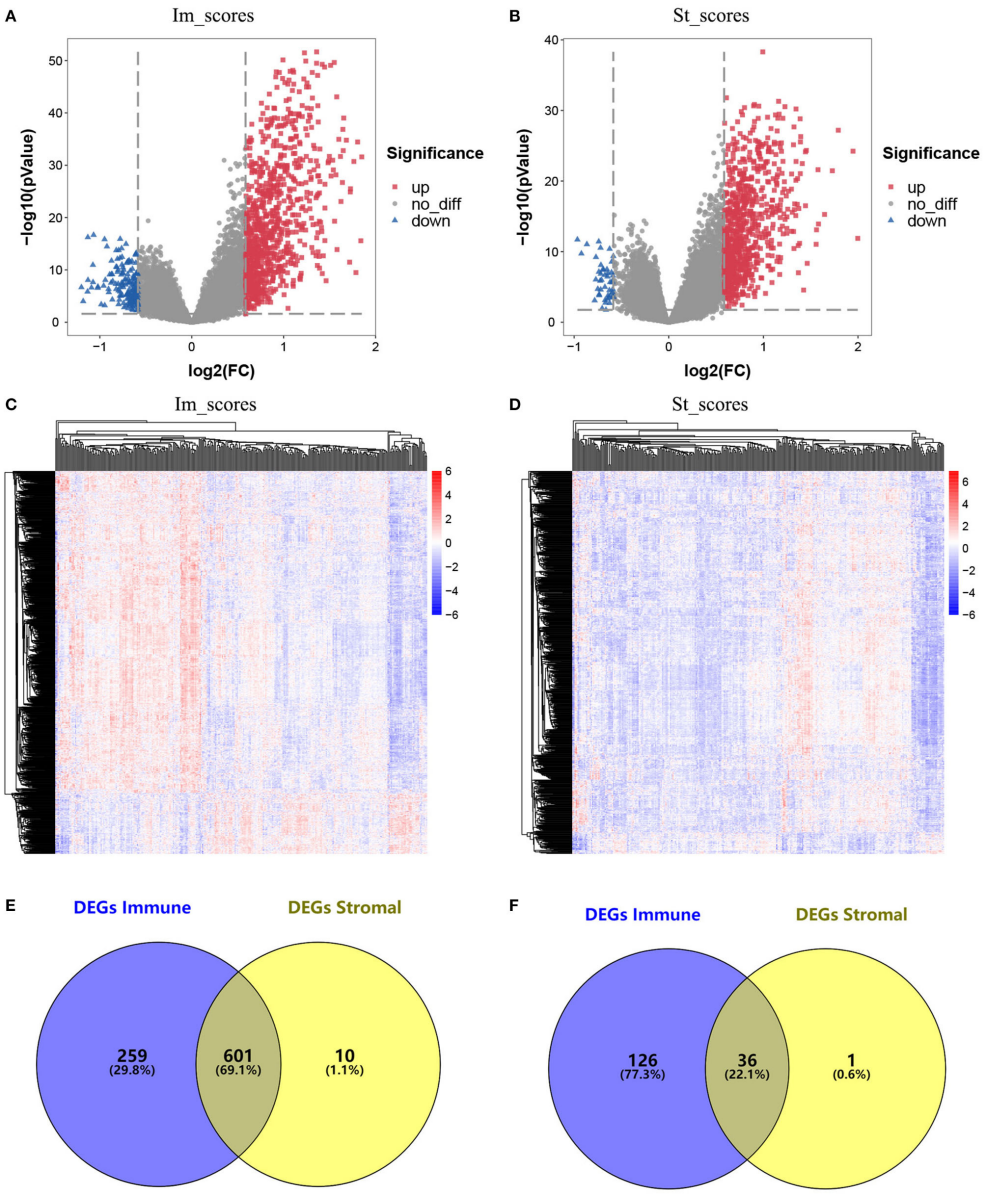
Comparison of gene expression profile with immune scores and stromal scores in glioblastoma (GBM). **(A)** Volcano plot of differentially expressed genes (DEGs) of immune scores. Red, upregulated DEGs; blue, downregulated DEGs. **(B)** Volcano plot of DEGs of stromal scores. Red, upregulated DEGs; blue, downregulated DEGs. **(C)** Heatmap of the DEGs of immune scores of top half (high score) vs. bottom half (low score). *p* < 0.05, fold change > 1.5). **(D)** Heatmap of the DEGs of stromal scores of top half (high score) vs. bottom half (low score). *p* < 0.05, fold change > 1.5). **(E,F)** Venn diagrams showing the number of commonly upregulated **(E)** or downregulated **(F)** DEGs in stromal and immune score groups.

The data obtained from TCGA database were standardized using the LIMMA package, and the results were visualized on heatmaps and a volcano map (Figures 2A–D). The analysis revealed different gene expression profiles for high/low immunoassays and high/low stromal assays. Subgroup analysis based on immune scores revealed that 860 genes were upregulated and 162 genes were downregulated in the high-score group compared with the low-score group (fold change ≥1.5, *p* ≤ 0.05). Similarly, 611 genes were upregulated and 37 were downregulated in the high stromal score group. The overlap between genes revealed that 601 were upregulated and 36 downregulated (Figures 2E,F). Interestingly, the DEGs extracted from the immune score comparison were found to have covered most of the genes extracted from the comparison based on stromal scores. Therefore, these DEGs were further analyzed.

**Figure 3:**
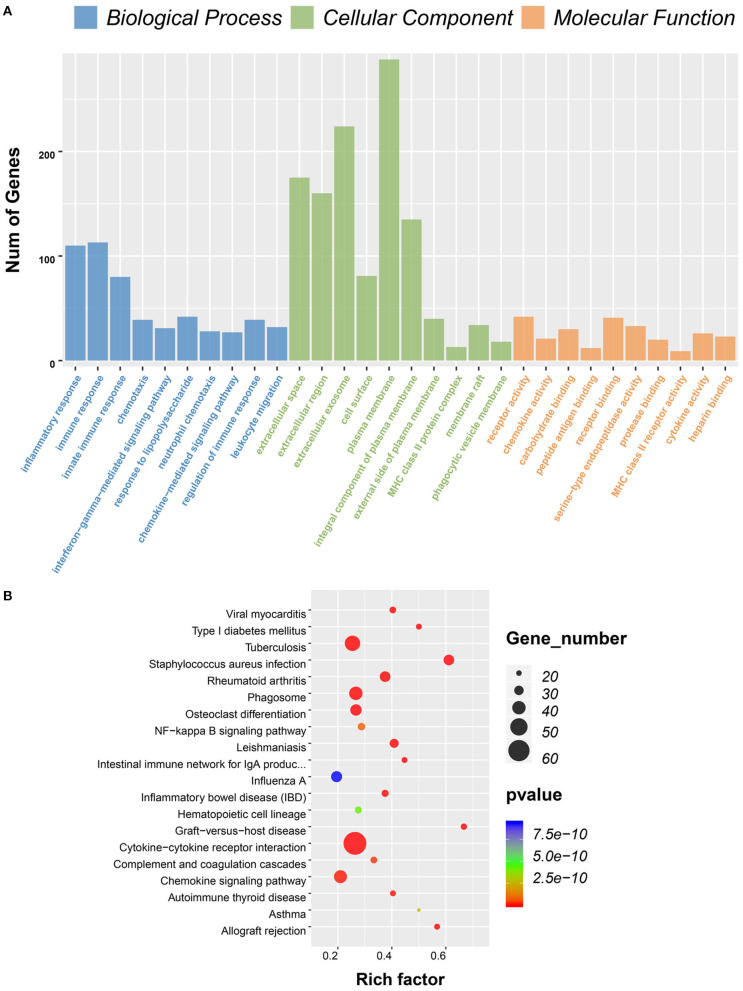
Gene ontology (GO) term and Kyoto Encyclopedia of Genes and Genomes (KEGG) pathway analysis for differentially expressed genes (DEGs) significantly associated with immune scores. **(A)** Top 10 GO terms. Number of gene of GO analysis was acquired from Database for Annotation, Visualization and Integrated Discovery (DAVID) functional annotation tool. *p* < 0.05. **(B)** KEGG pathway.

To predict the roles of the DEGs, GO function analysis was performed. For the immune score group, the DEGs were enriched in immune response in the biological component, inflammatory response in the cellular component, and plasma membrane and receptor activity in the MF component (Figure 3A). KEGG pathway analysis identified 57 pathways associated with the DEGs. The top 5 enriched pathways were cytokine–cytokine receptor interaction pathways (59 DEGs), viral protein cross-talk with cytokine and cytokine receptor pathways (34 DEGs), hematopoietic cell lineage (31 DEGs), chemokine signaling pathways (31 DEGs), and tuberculosis-related pathways (57 DEGs) (Figure 3B).

### Impact of the Differentially Expressed Genes on Overall Survival

**Figure 4:**
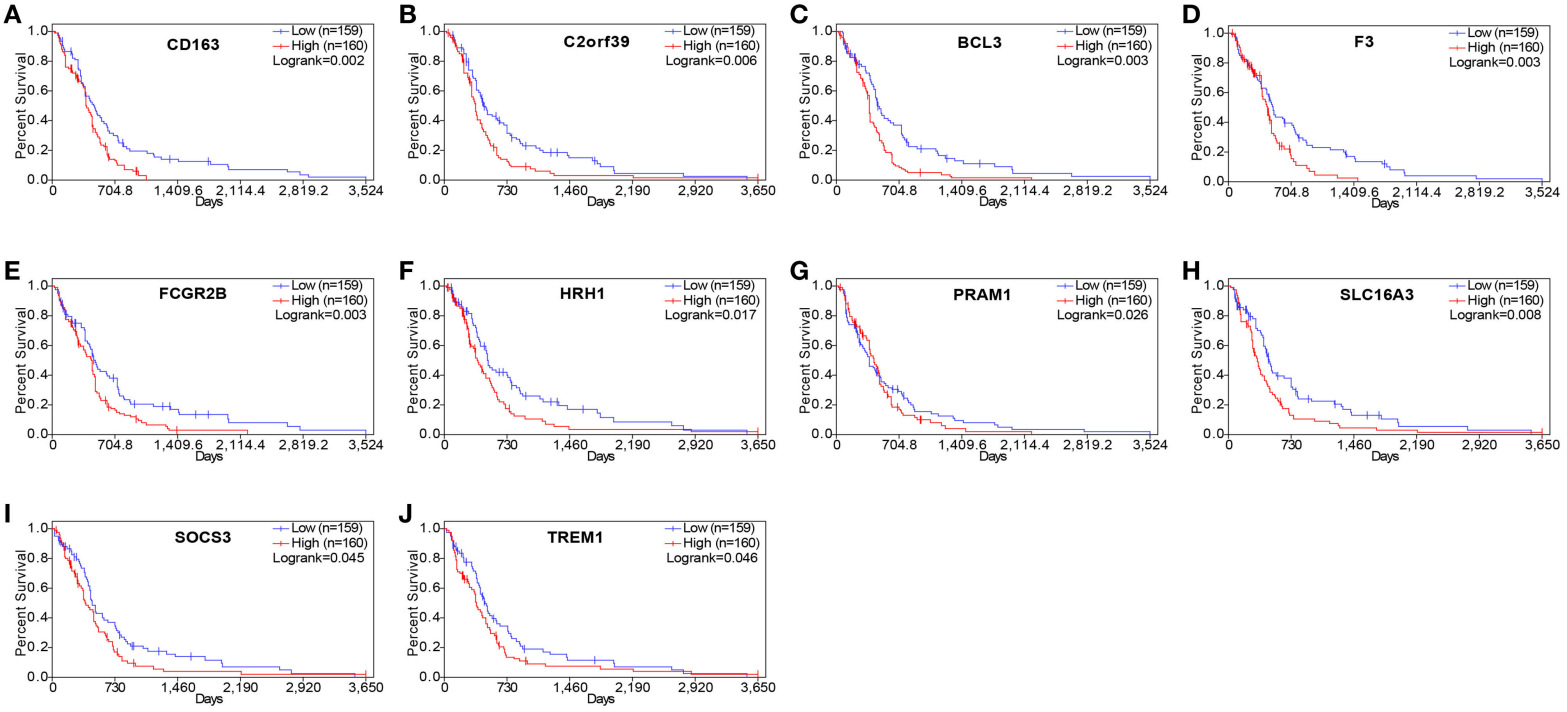
Correlation of expression of individual differentially expressed genes (DEGs) in overall survival in The Cancer Genome Atlas (TCGA). Kaplan–Meier survival curves were generated for selected DEGs extracted from the comparison of groups of high (red line) and low (blue line) gene expression. *p* < 0.05 in log rank test. OS, overall survival in days.

To determine whether the DEGs were associated with the OS, KM survival curves were designed based on TCGA dataset. Among the 860 DEGs upregulated in the high-immune scores group, 228 DEGs were significantly associated with poor OS (log-rank test, *p* ≤ 0.05, Table 1). The selected genes are shown in Figure 4.

**Table 1:** Two hundred twenty-eight significantly gene correlated with poor overall survival.

**Gene symbols**
*ABCC3, CASP4, DDIT4L,GPR84, LOC654346, PDPN, SDC2, TMEM176A, AHNAK2, CASP5c, DENND2D, GZMB, LOX, PHLDA3, SERPINA3, TMEM176B,* *ALPK1, CAST, DIRAS3 HCST, LOXL1, PKIB, SERPING1, TNC, ANG, CCDC109B, DKFZP586H2123, HLA-DPB2, LRG1, PLA2G5, SIGLEC10, TNFRSF11B,* *ANXA1, CCR5, DOK3, HOXB4, LRRC25, PLAUR, SIGLEC7, TNFRSF1A, ANXA2, CD109, DPYD, HPS3, LTF, PLP2, SIGLEC8, TPP1, ANXA4, CD14, DRAM,* *HRH1, LY75, PLSCR1, SIGLEC9, TREM1, APOB48R, CD163, DTX3L, HSPA6, MAN1C1, PLTP, SIPA1, TRIM6, APOBEC3C, CD44, ECGF1, IBSP, MAOB,* *PLXDC2, SLAMF8, TRPM8, APOBEC3F, CD68, EHBP1L1, IFITM2, MARCO, POSTN, SLC11A1, TTC12, APOBEC3G, CD84, EMP3, IFITM3, MDFIC, PPM1M,* *SLC16A3, UGCG, AQP9, CEBPD, ETV7, IL13RA1, MGC24103, PQLC3, SLC47A2, UNC93B1, ARPC1B, CEBPE, F3, IL4I1, MGC7036, PRAM1, SNX10, UPP1,* *ARSJ, CFI, FAH, IL7, MOXD1, PSCD4, SOCS3, VAMP5, BCL3, CHI3L1, FAM129A, ISG20, MR1, PTRF, SP140, VASN, BIC, CHIT1, FBLN5, ITGA3, MRO, PTX3,* *SPI1, VDR, BST1, CLDN23, FCER1G, ITGAM, MSR1, RARRES2, SPP1, WIPF1, BST2, CLEC5A, FCGBP, ITGB4, MST150, RCSD1, SQRDL, WWTR1,* *C10orf10, CLEC7A, FCGR2B, ITGB5, MXRA5, RDH10, SRPX2, C17orf87, COL8A1, FCGR3A, KCNN4, MYBPH, RIN3, STAB1, C1orf34, COL8A2, FER1L3,* *LCTL, NAGA, RIPK3, STEAP3, C1RL, COPZ2, FES, LGALS1, NCF1, RNASE2, tcag7.1314, C1S, CP, FGL2, LGALS3, NFE2L3, RNASE3, TCTEX1D1, C21orf62,* *CPD, FLJ46266, LGALS8, NFKBIZ, RNASE4, TFEC, C2orf39, CSF3R, FMOD, LGALS9, NMI, S100A10, TGFBI, C3AR1, CTSB, FTL, LHFPL2, NYD-SP21,* *S100A11, THNSL2, C5AR1, CTSL1, FZD7, LILRB3, OCIAD2, S100A4, TIMP1, C5orf29, CXCL10, GJB2, LIMS1, P4HA2, SAMD9, TLR3, CA12, CXCL14,* *GPR160, LOC388335, PBEF1, SAMD9L, TMBIM1, CAPG, CYP19A1, GPR18, LOC493869, PDLIM4, SCIN, TMEM154*

### Construction of a Protein–Protein Interaction Network for the Genes With Prognostic Value

**Figure 5:**
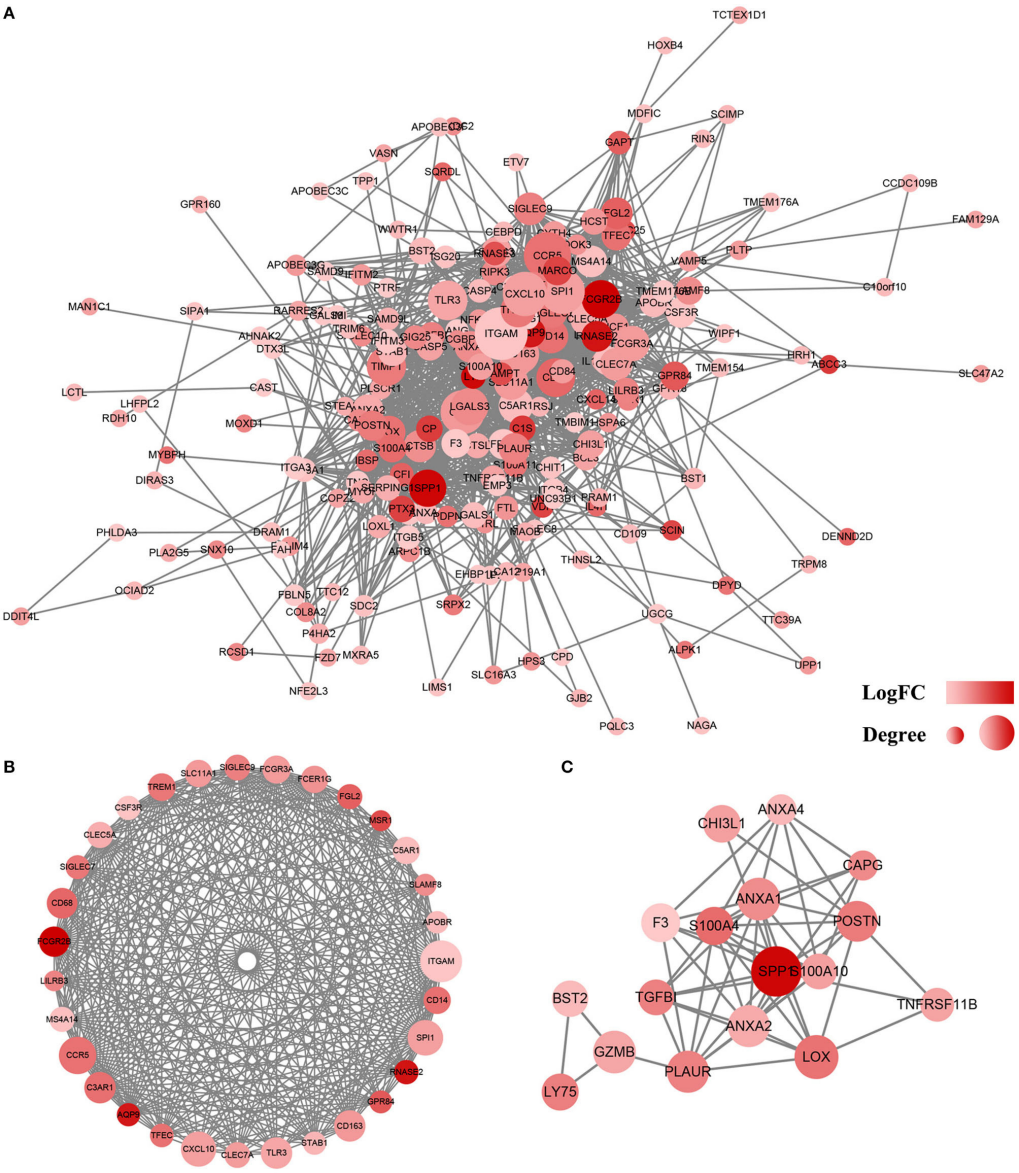
Protein–protein interaction (PPI) network of differentially expressed genes (DEGs). **(A)** Based on the STRING online database, 207 genes/nodes were filtered into the DEG PPI network. **(B)** The most significant module 1 from the PPI network. **(C)** The second significant module 2 from the PPI network. The color of a node in the PPI network reflects the log (FC) value of the Z score of gene expression, and the size of node indicates the number of interacting proteins with the designated protein.

To analyze the interaction between identified DEGs, a PPI network was constructed using the STRING (http://string-db.org). A total of 228 genes were included in the PPI network, which contained co-genes, including 207 nodes, 1,481 edges, and a score > 0.25 (Figure 5A). With the use of MCODE analysis, the top 2 significant modules were selected for further analysis (Figures 5B,C, Table 2).

**Table 2:** Forty-eight genes in the two modules that obtained from TCGA database.

**Categories**	**Gene symbols**
Cluster 1	*AQP9, SLC11A1, TLR3, SIGLEC9,* *SIGLEC7, TFEC, FGL2, ITGAM, CD14,* *CD68, LILRB3, CD163, CXCL10, CCR5,* *CSF3R, C5AR1, MSR1, TREM1,* *FCGR2B, FCER1G, FCGR3A, CLEC7A,* *C3AR1, SPI1, MS4A14, SLAMF8, GPR84,* *CLEC5A, RNASE2, APOBR, STAB1*
Cluster 2	*SPP1, S100A4, TGFBI, POSTN, LY75,* *CAPG, PLAUR, ANXA4, BST2, GZMB,* *S100A10, LOX, CHI3L1, TNFRSF11B,* *ANXA2, ANXA1, F3*

*TCGA, The Cancer Genome Atlas*.

### Functional Enrichment Analysis for Genes With Prognostic Value

**Figure 6:**
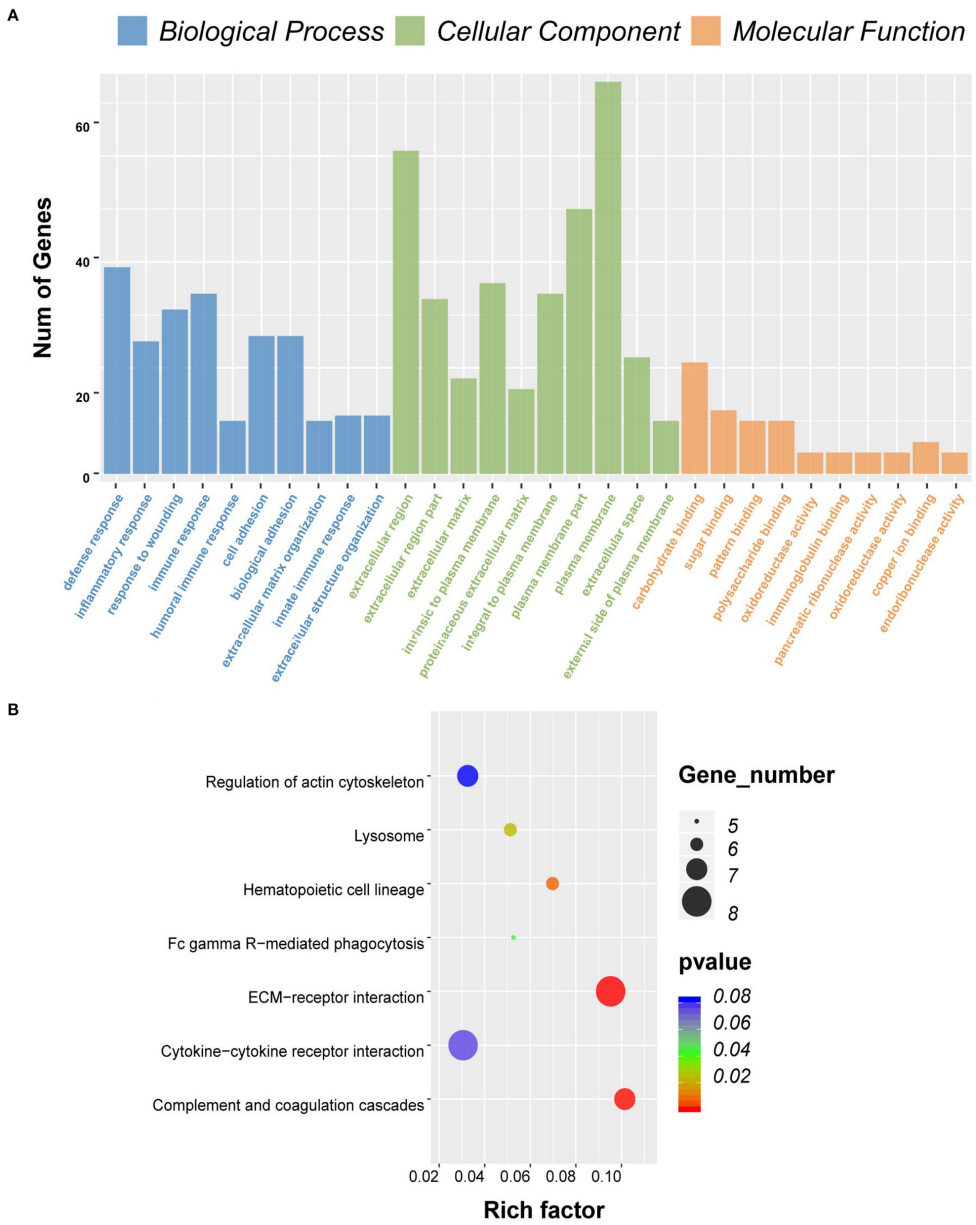
Gene ontology (GO) term and Kyoto Encyclopedia of Genes and Genomes (KEGG) pathway analysis for differentially expressed genes (DEGs) significantly associated with overall survival. **(A)** Top 10 GO terms. Number of gene of GO analysis was acquired from Database for Annotation, Visualization, and Integrated Discovery (DAVID) functional annotation tool. *p* < 0.05. **(B)** KEGG pathway.

Consistent with PPI network analysis, functional enrichment clustering of these genes revealed a strong association of the genes and immune response. A total of 115 GO terms of BP, 21 of CC, and 48 of MF were found to be significant (FDR ≤ 0.05, –log FDR ≥ 1.301). The top GO terms associated with the genes were immune/inflammatory response, extracellular region, and carbohydrate binding (Figure 6A). Moreover, KEGG analysis revealed that all pathways were associated with immune response (Figure 6B).

### Validation of the Prognostic Value of Genes in the Chinese Glioma Genome Atlas Dataset

**Figure 7:**
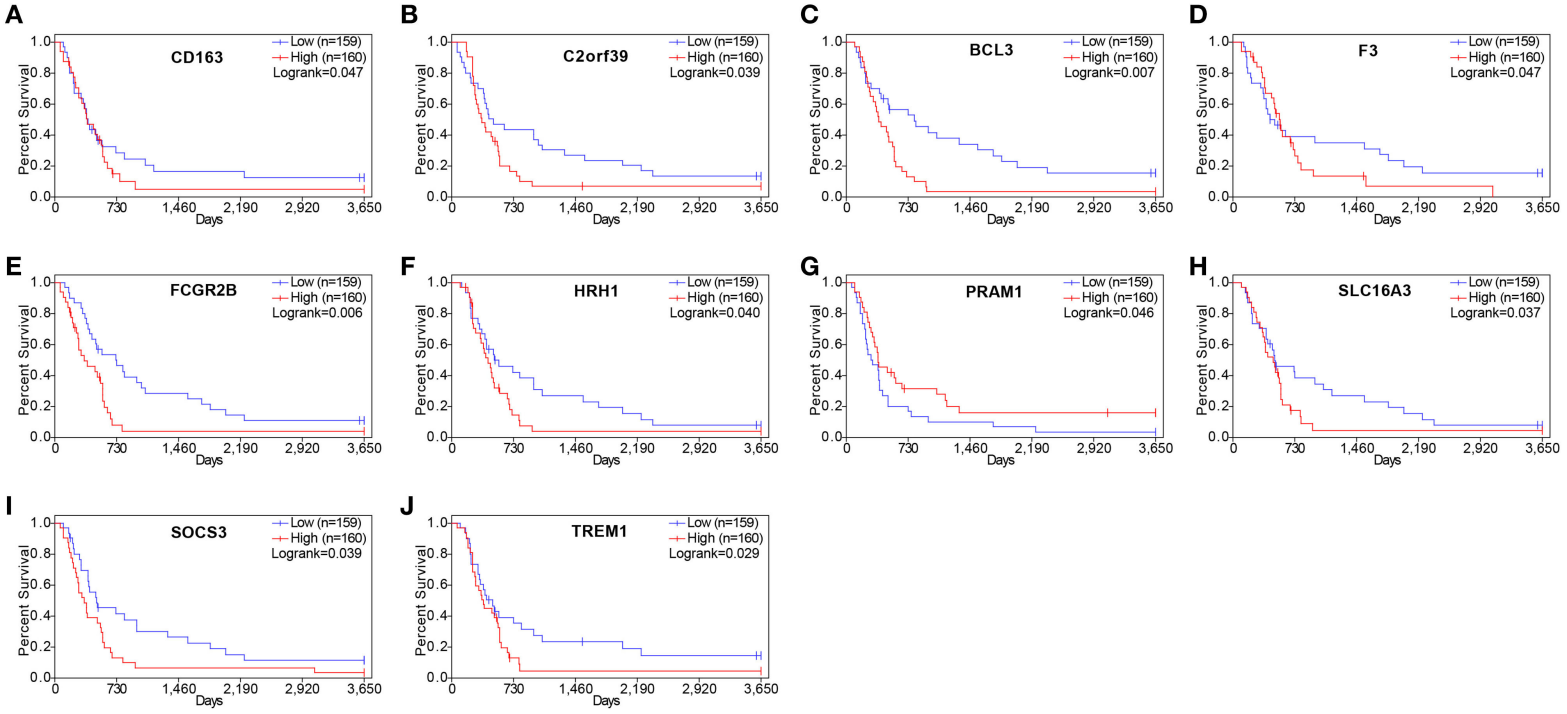
Validation of correlation of differentially expressed genes (DEGs) extracted from The Cancer Genome Atlas (TCGA) database with overall survival in Chinese Glioma Genome Atlas (CGGA) cohort. Kaplan–Meier survival curves were generated for selected DEGs extracted from the comparison of groups of high (red line) and low (blue line) gene expression. *p* < 0.05 in log rank test. OS, overall survival in days.

To understand whether the genes identified in TCGA database also affect the prognosis of other cases of GBM, we downloaded and analyzed gene expression data from 124 cases of GBM in an independent glioma database, CGGA. A total of 10 genes were validated (Figure 7) to be significantly associated with poor prognosis (Table 3).

**Table 3:** Genes significant in GBM overall survival identified in both TCGA and CGGA.

**Gene symbols**
*BCL3, C2orf29, CD163, F3, FCGR2B, HRH1, PRAM1, SLC16A3, SOCS3, TREM1*

*GBM, glioblastoma; TCGA, The Cancer Genome Atlas; CGGA, Chinese Glioma Genome Atlas*.

### Drug–Gene Interaction and Functional Analysis of the Genes

Drug–gene interaction analysis was performed on the 10 validated genes assigned to the critical gene module 1. The DGIdb analysis identified 186 drugs that interacted with the histamine receptor H1 (*HRH1*) gene, 22 drugs that interacted with Fc fragment of IgG receptor IIb (*FCGR2B*) gene, two drugs that interacted with solute carrier family 16 members 3 (SLC16A3), two drugs that interacted with CD163 (CD163 molecule), and three drugs that interacted with coagulation factor III, tissue factor (F3). Of the 215 drugs, 14 targeted FCGR2B and SLC16A3 and exhibited antineoplastic activity and anti-GBM activity (Table 4).

**Table 4:** Candidate drugs targeting genes with glioblastoma.

**Number**	**Drug**	**Gene**	**Drug–gene interaction**
1	Cetuximab	FCGR2B	Antineoplastic
2	Etanercept	FCGR2B	Antineoplastic
3	Adalimumab	FCGR2B	Antineoplastic
4	Trastuzumab	FCGR2B	Antineoplastic
5	Rituximab	FCGR2B	Antineoplastic
6	Muromonab-CD3	FCGR2B	Antineoplastic
7	Tositumomab	FCGR2B	Antineoplastic
8	Alemtuzumab	FCGR2B	Antineoplastic
9	Alefacept	FCGR2B	Antineoplastic
10	Efalizumab	FCGR2B	Antineoplastic
11	Daclizumab	FCGR2B	Antineoplastic
12	Bevacizumab	FCGR2B	Antineoplastic
13	Natalizumab	FCGR2B	Antineoplastic
14	Streptozotocin	SLC16A3	Antineoplastic

## Discussion

**Figure 8:**
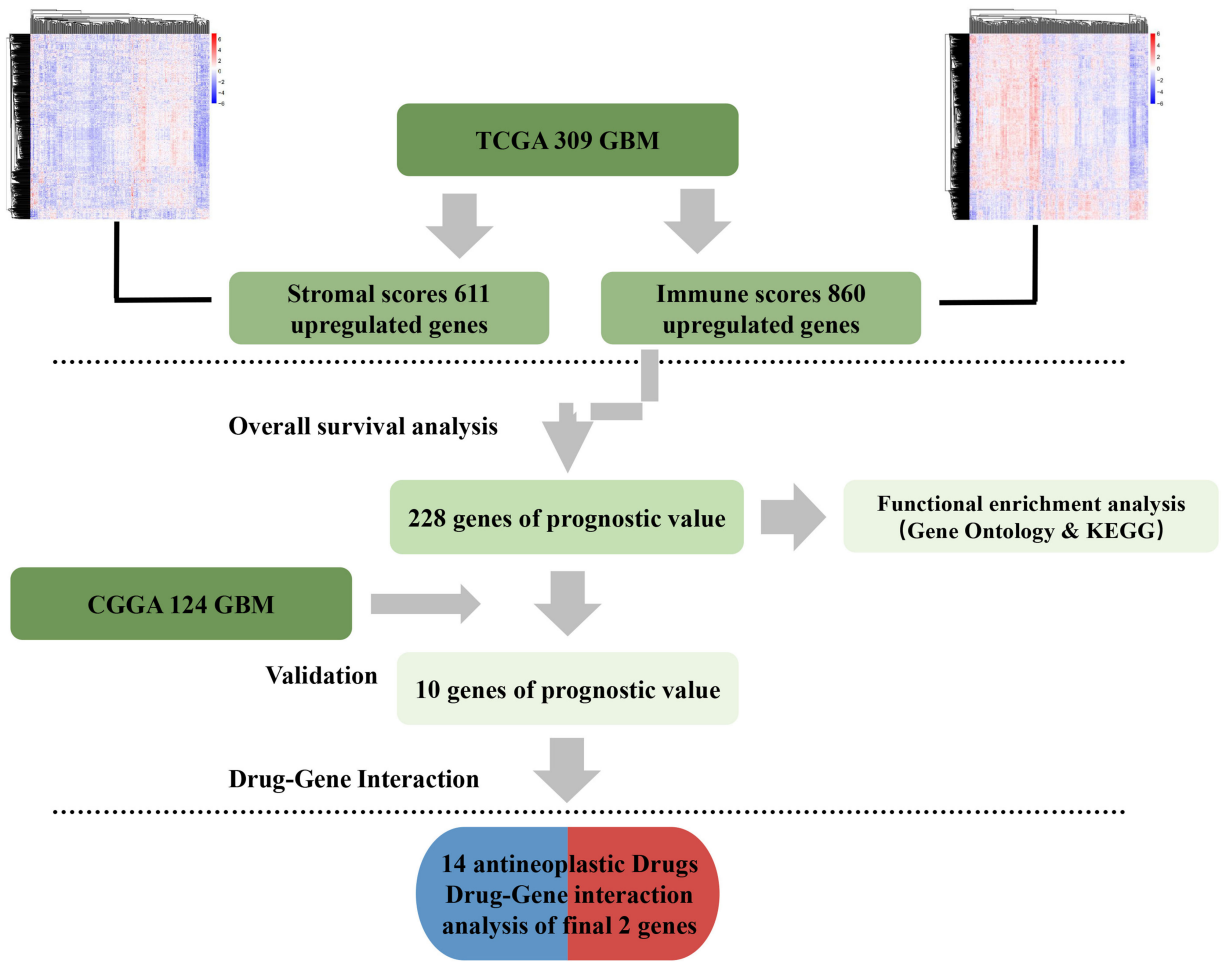
Data analysis workflow.

Here, gene expression data from TCGA and survival data of 309 GBM cases were analyzed. A total of 228 genes were found to be differentially expressed between samples with high and low immune or stroma scores. Importantly, 10 genes were validated in GBM patients from CGGA, a separate GBM database (Figure 8). Finally, analysis of gene–drug database identified 14 drugs that interacted with the 10 genes.

Comparison between high and low immune scores groups identified 860 DEGs, many of which were found to be involved in the TME, as revealed by GO term analysis (Figure 3). This finding was consistent with previous reports on the function of immune cells and extracellular matrix (ECM) molecules in the TME of GBM (30–33).

Next, we examined the impact of the 860 genes on the OS of GBM patients. The results showed that 228 genes were associated with poor outcomes. Additionally, we constructed the top 2 PPI modules that were related to immune/inflammatory responses (Figure 5).

Finally, using an independent cohort of 124 GBM patients, the 10 TME-related genes were validated and found to be significantly correlated with the prognosis of GBM (Table 3). Analysis of the DGIdb database identified 215 drugs that were associated with the genes. Among them, 14 drugs targeted FCGR2B and SLC16A3 genes and exhibited antineoplastic properties.

SLC16A3 regulates the secretion of lactic acid, which maintains pH and the Warburg effect (34). Multiple studies indicate that overexpression of SLC16A3 promotes the growth and proliferation of hypoxic cancer cells and is associated with poor cancer prognosis (35–37). FCGR2B belongs to the rhodopsin-like G-protein-coupled receptor family. It is expressed in multiple diseases, including systemic lupus erythematosus, non-small cell lung cancer, and IgA nephropathy liver hepatocellular carcinoma (38–42).

The expression of genes has been reported to influence the OS of GBM, based on findings from cancer cell line experiments, animal tumor models, or patient samples. However, due to the complexity of the GBM microenvironment, a more in-depth analysis should be performed using larger patient cohorts. The rapid development of whole-genome sequencing technology has led to the development of high-throughput, publicly available cancer databases, such as TCGA and CGGA. Other databases such as DGIdb provide a basis for large GBM data analysis (8, 43–45).

Previous studies have found that various tumor intrinsic genes influence various aspects of the TME (8). Here, we focused on gene features of the TME that influence the development, progression, and the OS of GBM patients. The findings of this study expand our understanding of the complex interaction between GBM and its TME.

In summary, this study found 10 TME-related genes that influence the OS of GBM patients. By using the DGIdb database, several drugs that interact with the genes in GBM were identified. Some of the identified genes can be considered as potential biomarkers of GBM. It would be interesting to find out whether a combination of these genes can better predict the survival rates of GBM patients. Further characterization of the identified drugs is advocated to reveal effective drugs for the treatment of GBM.

## Data Availability Statement

Publicly available datasets were analyzed in this study. This data can be found at: https://tcga-data.nci.nih.gov/tcGA/.

## Author Contributions

ZJ, BZ, YZ, and YS conceived and designed this study. ZJ wrote this manuscript. ZW revised this manuscript. ZJ made these figures with the help of YS, YX, ZW, and GT. All authors contributed to the article and approved the submitted version.

## Conflict of Interest

The authors declare that the research was conducted in the absence of any commercial or financial relationships that could be construed as a potential conflict of interest.
